# Juvenile African Clawed Frogs (*Xenopus laevis*) Express Growth, Metamorphosis, Mortality, Gene Expression, and Metabolic Changes When Exposed to Thiamethoxam and Clothianidin

**DOI:** 10.3390/ijms222413291

**Published:** 2021-12-10

**Authors:** Jill A. Jenkins, Katherine R. Hartop, Ghadeer Bukhari, Debra E. Howton, Kelly L. Smalling, Scott V. Mize, Michelle L. Hladik, Darren Johnson, Rassa O. Draugelis-Dale, Bonnie L. Brown

**Affiliations:** 1 U.S. Geological Survey, Wetland and Aquatic Research Center, 700 Cajundome Boulevard, Lafayette, LA 70506, USA; johnsond@usgs.gov (D.J.); daler@usgs.gov (R.O.D.-D.); 2 Department of Biology, Virginia Commonwealth University, Richmond, VA 23284, USA; khartop@gmail.com (K.R.H.); gfbukhari@kau.edu.sa (G.B.); pridgende@vcu.edu (D.E.H.); bonnie.brown@unh.edu (B.L.B.); 3 U.S. Geological Survey, New Jersey Water Science Center, Lawrenceville, NJ 08648, USA; ksmall@usgs.gov; 4 U.S. Geological Survey, Lower Mississippi-Gulf Water Science Center, Baton Rouge, LA 70816, USA; svmize@usgs.gov; 5 U.S. Geological Survey, California Water Science Center, 6000 J Street, Placer Hall, Sacramento, CA 95819, USA; mhladik@usgs.gov

**Keywords:** *Xenopus laevis*, metamorphosis, neonicotinoids, liver enzymes, gene expression, biomarkers, flow cytometry

## Abstract

Neonicotinoids (NEO) represent the main class of insecticides currently in use, with thiamethoxam (THX) and clothianidin (CLO) primarily applied agriculturally. With few comprehensive studies having been performed with non-target amphibians, the aim was to investigate potential biomarker responses along an adverse outcome pathway of NEO exposure, whereby data were collected on multiple biological hierarchies. Juvenile African clawed frogs, *Xenopus laevis*, were exposed to commercial formulations of THX and CLO at high (100 ppm) and low (20 ppm) concentrations of the active ingredient. Mortality, growth, development, liver metabolic enzyme activity, and gene expression endpoints were quantified. Tadpoles (*n* > 1000) from NF 47 through tail resorption stage (NF 66) were exposed to NEO or to NEO-free media treatments. Liver cell reductase activity and cytotoxicity were quantified by flow cytometry. Compared to control reference gene expressions, levels of expression for NEO receptor subunits, cell structure, function, and decontamination processes were measured by RT-qPCR by using liver and brain. Mortality in THX high was 21.5% compared to the control (9.1%); the metabolic conversion of THX to CLO may explain these results. The NF 57 control tadpoles were heavier, longer, and more developed than the others. The progression of development from NF 57–66 was reduced by THX low, and weight gain was impaired. Liver reductases were highest in the control (84.1%), with low NEO exhibiting the greatest reductions; the greatest cytotoxicity was seen with THX high. More transcriptional activity was noted in brains than in livers. Results affirm the utility of a study approach that considers multiple complexities in ecotoxicological studies with non-target amphibians, underscoring the need for simultaneously considering NEO concentration-response relationships with both whole-organism and biomarker endpoints.

## 1. Introduction

Amphibian population declines and morphological malformations recorded globally since the early 1990’s have been attributed to multiple stressors, whereby habitat loss associated with agricultural expansion is considered the predominant activity affecting lowland amphibian populations [1,2,3,4]. Subsequent to habitat loss, pollutants such as anthropogenic chemicals are the next major influence on amphibians [5]. Studies on the direct effects of pesticides on amphibian growth and survival are more routine than those conducted on indirect effects [6,7], yet even those studies are complicated with confounding aspects such as animal densities, proximity to the chemicals, forage, and predators. Results on the indirect biological or sublethal effects can show longer larval periods, developmental abnormalities, increased susceptibility to disease and predation, slowed swimming, as well as genotoxic and cytotoxic effects after concentration exposures well below those associated with mortality [8,9,10,11,12,13]. Although rarely considered in risk assessments, the indirect effects of agrochemicals are essential in delineating an adverse outcome pathway (AOP) framework for ecosystem integrity studies integral to amphibian conservation efforts [10,14,15]. An AOP is a conceptual construct that portrays existing knowledge concerning the linkage between a direct molecular initiating event and a resultant adverse outcome at particular biological level of organization [14].

Neonicotinoids (NEO) are urban- and agriculturally applied neurotoxic pesticides that have become the most widely used insecticide class worldwide, generally considered of low toxicity to vertebrates and as replacements for many in other insecticide classes [16,17,18,19,20]. With high water solubility, widespread application, and environmental persistence [21,22], NEO are detected frequently in aquatic ecosystems (0.30–0.34 g L^−1^ for CLO and 4.1 g L^−1^ for THX) [19,23,24,25,26,27]. Multiple NEO applications and subsequent agrochemical runoffs over a growing season can expose an amphibian at multiple time points during its ontogenesis [28]. Mixtures of fungicides and insecticides are able to persist in frog tissues at concentrations up to 1.5 mg kg^−1^ wet weight [2]. NEO can occur in waters at breeding sites, as run-off in puddles and ephemeral ponds, and in agricultural fields, with tadpoles being particularly susceptible due to their permeable skin and often living at the aquatic and terrestrial interface [2,29,30,31].

Physiological responses of amphibians vary by animal age and developmental stage [6,28,32,33]. For example, hepatic processing of polyaromatic hydrocarbons is less complete in larval *Xenopus tropicalis* than in adults [34], yet the hypothalamo–pituitary–interrenal axis of premetamorphic *Rana pipiens* and *Xenopus laevis* is more responsive to external stressors than in the later developmental stages [35]. After metamorphosis, amphibian lymphocyte numbers expand rapidly and immune defenses mature, yet the early juvenile period is a likely time of vulnerability to disease [36]. The conserved neuroendocrine stress axis mediates the response of the animal to its environment, with bi-directional communication between the immune system and the nervous system [36]. Prolonged environmental stressors, such as chronic or pulsating exposures to NEO, may induce chronic release of corticosteroids, with corticosterone (the main corticosteroid hormone in amphibians) being elevated during metamorphosis [37].

Species-specific responses can be induced by pesticides and their formulations [6,38,39]. Organismal mortality from pesticides is typically measured as the LC_50_, or the concentration in water which is lethal to 50% of the test organisms; too few species are typically assessed to reflect the true variation in responses from the array of exposed species in the wild [10,40]. Sublethal effects of NEO in fish, and likewise amphibians, in aquatic habitats have not yet been sufficiently studied [41,42]. In mammals, a difference in response to THX was shown in mice, but not rats, that THX is hepatotoxic and hepatocarcinogenic, with the murine liver cytochrome P450 (Cyp) enzymes being more active in generating formaldehyde from THX [20,43].

Pesticides generally initiate at a known molecular site of action [44]. The NEO bind to nicotinic acetylcholine receptors (nAChR), a pentameric transmembrane complex consisting of diverse subunits [19,45]. Receptors for NEO occur on cells in organisms throughout the animal kingdom [10] and on various tissues within organisms [45], with NEO binding being less effectual in animals other than insects due to nAChR molecular conformation differences [19]. The NEO and their metabolites are agonists of the neurotransmitter acetylcholine [46], with binding generating a continuous excitatory nervous signal leading to neurotoxic death in insects.

After binding by a ligand, receptors exert cellular functions with various outcomes. In a reptilian model, THX directly increased the concentrations of brain acetylcholine by upregulating the expression of *ache* [47]. This nAChR blockage by NEO ligands triggers increased production of acetylcholinesterase (AChE) that hydrolyzes acetylcholine into choline and acetic acid; such an AChE increase was shown in honeybees *Apis mellifera* and water fleas *Daphnia magna* exposed to NEO. Changes in AChE levels have long been used as biomarkers in fish for organophosphorus (OP) insecticide exposure [48,49,50]. While acetylcholine is perceived as a neurotransmitter, the presence of cholinergic systems on many nonneuronal tissues and cells (including sperm) demonstrates its use as a local signaling molecule [45]. Chronic exposure to NEO upregulates vertebrate α4β2 subunit nAChR, and rat α4β2 and α7 of nAChR subtypes, as well as muscarinic cholinergic receptors (mAChR) involved with CLO exposure [51,52]. Understanding of these receptor types continues to evolve with time; in general, muscarinic receptors occur at neuromuscular junctions and nicotinic receptors are synaptic, with much of the pioneering research being performed in amphibians [53].

In amphibians, liver enzymatic mechanisms and gene expressions have been used as biomarkers of exposure [54,55,56]. Metabolism of NEO, as well as the development of NEO resistance in target-pests, is modulated by metabolic detoxifying enzymes from the cytochrome P450-monooxygenases (P450), glutathione transferases, and carboxylesterases. In mammals, NEO are metabolized through oxidation reactions mediated by cytochrome P450 19, and in human cell lines, non-monotonic concentration response curves were shown by NEO-induced cytochrome P450 19 gene expression with a decline in gene induction and catalytic activity at the higher concentrations [57]. Metabolism of NEO either increases or decreases its potency depending on the compound and the specificity of the nAChR, with the CYP enzymes orchestrating a multitude of reactions [58,59].

As the United States Environmental Protection Agency (USEPA) has scheduled NEO regulatory reviews for completion in 2022, the data generated here address knowledge gaps in this complex ecotoxicological arena. Our hypothesis was that effects along an AOP in *Xenopus laevis* may occur with NEO exposure during metamorphosis. The goal was to characterize potential sublethal effects at various levels of biological hierarchy, including survival, larval growth and development, liver metabolism, and transcription of relevant genes. As a model species, the fully aquatic *X. laevis* is easily maintained in the laboratory, and ample curated literature resources are available [60]. Dermal contact is the major route of exposure to NEO for amphibians in aquatic stages, thus understanding if commercial formulations of THX and CLO induce morphological, cellular, or molecular changes under controlled laboratory settings is relevant for this taxon of conservation focus, adding interpretations to field study results.

## 2. Methods

### 2.1. Animals and Husbandry

This research was carried out via taxon-approved guidelines [61,62] through Virginia Commonwealth University (VCU) approval of the Institutional Animal Care and Use Committee protocol AD20261. Developmental staging by Nieuwkoop and Faber (NF) was used to describe juvenile development [63], with careful dorsal side observations of morphological changes, including limb bud development. *Xenopus laevis* larvae were obtained by in vitro fertilization using standard methods [64] in a VCU embryology laboratory. Briefly, eggs from 3 females were collected in 0.1× Modified Barth’s Saline (MBS, pH 7.8) [65] and fertilized by using sperm obtained from 2 males. Embryos were cultured in MBS in 15 cm diameter petri dishes (2.2–3.4 cm^3^ per tadpole) and incubated (Torrey Pines Scientific, Cat. No. IN30) at 23 °C for six days after fertilization through stage NF 44. Unused embryos were given a lethal dose of anesthetic (10% tricaine). Immediately after hatch-out, larvae were cultured in 0.1× MBS control without pesticide or with NEO in 0.1× MBS, with daily refreshing. The containers were bisphenol A-free plastic (P5) 1-L containers for stages NF 47–66. These final 13 × 30 cm containers provided 205–278 cm^3^ per tadpole. Tadpoles were held at as similar densities as possible (Supplementary Table S1) to limit density-specific growth effects [66]. The spatial order of vivaria was rearranged periodically to minimize possible effects of location and light. For the duration of the experiment, tadpoles experienced a daily photoperiod 12:12 h light to dark with filtered low-light conditions to eliminate the potential for NEO photodegradation [22,67]. Sufficient oxygen was delivered with aerators, generating continual air flow through the center of each vivarium. Tadpoles were fed twice daily with 2 mL of a liquid nutrient suspension (Supplementary Table S2). Larvae were cultured through metamorphic climax until tail resorption.

### 2.2. Exposures and Biological Endpoints

Thresholds for NEO in amphibians have not yet been established, but LC_50_ levels for some fish species have been published (Table 1) and were thus used as the basis for the exposure concentrations selected in this study. For USEPA risk estimations, when chemical-specific data are not available from the agency or the open literature, as in the case of amphibians and NEO, the Agency relies on fish data as surrogates for aquatic phase amphibians [44,68]. Fish are typically less sensitive to NEO than insects or crustaceans, with fish acute median lethal and effect concentrations (LC_50_/EC_50_) observed to be >80 mg L^−1^, exceeding surface water exposure concentrations [42,68]. Thus, to explore uncertainties associated with the absence of NEO toxicity data available for non-target amphibians, the concentrations of CLO and THX were selected based on fish LC_50_ values [44,68,69,70,71,72] (Table 1). In accord with USEPA guidance for addressing non-definitive acute and sub-acute toxicity endpoints [73], levels above estimated environmental concentrations were tested to examine sublethal effects on tadpoles. The CLO and THX exposures were performed at 20 ppm and 100 ppm, higher than the typical sub-lethal concentrations in the wild [16,74,75]. Moderate mortality in adult wood frogs was induced by THX at 38 mg L^−1^ [76]. This current chronic exposure study considered that NEO effects are cumulative with time [77], that the ecological thresholds for NEO in water were published as 0.2 µg L^−1^ (short-term acute) or 0.035 µg L^−1^ (long-term chronic) [16], and that the exposure concentrations for fish are listed [78]. At day 21, aqueous concentrations of NEO were confirmed via liquid chromatography tandem mass spectrometry [79] (Supplementary Table S3).

The exposure period spanned pre-metamorphic development (NF 47–57) until day 44 with growth and development being tracked [80]. Each treatment consisted of NEO formulated for agricultural applications containing TMX or CLO, both at either 100 ppm or 20 ppm (referred to as high or low concentrations), or a NEO-free MBS control. The commercial formulations were Belay^®^ (Valent, Walnut Creek, CA, USA) that contained the active ingredient (a.i.) CLO (CAS registry number 210880-92-5 at 22.3–23.7% a.i.) and Platinum 75 SG^®^ (Syngenta, Greensboro, NC, USA) that contained THX a.i. (CAS registry number 153719-23-4 at 75% a.i., Supplementary Table S4). Dosing occurred every 24 h by complete solution change with chemical renewal. For each of the 5 treatments, 4 replicate vivaria were employed, with each vivarium initially housing approximately 50 tadpoles (Supplementary Table S1). Tadpole lengths were measured after photographing on days 5, 19, 30, and 44. Total lengths, wet weights, and stage were measured on day 44. Tadpoles were sampled for liver metabolism and genomic biomarker endpoints at NF 47–57 and NF 57, respectively [81].

From NF 57 to tail resorption stage at NF 66 (day 76) or complete metamorphosis, a second exposure was performed similarly by using 80 randomly chosen tadpoles from respective first exposures. Each of the 4 replicate vivaria per treatment group was stocked with 5 tadpoles, except for CLO high having had only two replicates due to low numbers of remaining tadpoles after the first exposure. Each tadpole was observed daily for 33 days, and the developmental stage was recorded (NF 57 to 66). Because individuals were not physically marked or tagged, staging scores were recorded per treatment group. All mortalities were recorded over the course of the exposures [80].

### 2.3. Dissection

Tadpoles were dissected after sedation with 0.04 mM tricaine methanesulfonate, pH 7.4 (Sigma Aldrich, St. Louis, MO, USA) [82]. For gene expression, dissection tools, pins, and plates were pre-cleaned using 1 M HCl followed by 20% chloroform and a nucleotide-free water wash. Tadpoles pinned to a dissection plate remained sedated while liver and brain tissues were removed (Supplementary Figure S1) and placed into 800 µL ice-cold TRIzolTM Reagent (ThermoFisher Scientific, Waltham, MA, USA). Tissues were homogenized by using sterile BioMasher II^®^ (Kimble Chase Life Science and Research Products, LLC, Vineland, NJ, USA), weighed and stored at −80 °C (See Supplementary Materials).

### 2.4. RNA Extraction

Surfaces and equipment were RNase-decontaminated with RNaseZAP^®^ (cat. #AM9780; ThermoFisher Scientific, Waltham, MA, USA). Liver and brain tissue messenger RNA (mRNA) was extracted and purified in triplicate per animal by using the TRIzol^®^ Reagent protocol [83]. The aqueous phase was extracted by using 200 µL chloroform with a 5 min incubation at 24 °C. Genomic DNA (gDNA) was removed by using 7.5 μM lithium chloride (ThermoFisher Scientific, Waltham, MA, USA) precipitation at −20 °C overnight.

### 2.5. Primer Development, Quantitative Real Time Polymerase Chain Reaction (qrtPCR) Assays, and Gene Expression Analysis

Gene sequences for primer design were selected from XenBase (http://www.xenbase.org/, RRID:SCR_003280; accessed on 17 June 2016) [60] and NCBI GenBank (https://www.ncbi.nlm.nih.gov/genbank/; accessed on 17 June 2016) [84]. Primer sequences were 18–20 bp in length, having a PCR melting temperature of ~60 °C, high GC content, thus resulting in a product ~150 bp by using the Primer3Plus algorithm (version 2.4.0) [85,86]. By having the amplicon span an exon-exon boundary, primers would have minimal gDNA contamination. Visual screening for gDNA contamination in the RNA preparations was performed during assay development by using TapeStation 2200 (Agilent, Santa Clara, CA, USA). Correct quantitative real-time reverse transcription PCR (qrtPCR) amplification of the target genes was confirmed by using Sanger Sequencing (Genewiz; Boston, MA, USA; see Sequencing section). Because searches did not yield results for gene transcription on *X. laevis* combined with CLO and THX, the reference gene primer sets selected were those applied in *X. laevis* developmental studies [87] and complemented those designed in this study (Table 2).

Primer utility was assessed by examining the standard curve and confirmed by using PCR reactions. For the standard curve method, a serial dilution of stock cDNA was used to perform qPCR and the subsequent melt curve. Multiple peaks in the melt curve were used to assess non-specific amplicon formation. This was then confirmed using PCR (Apex Hot-start Taq ™ El Cajon, CA, USA) for 15 min at 95 °C, followed by 35 cycles of 30 sat 95 °C, specific to primer temperature, and 30 s at 72 °C, with a final incubation for 5 min at 72 °C. Products were electrophoresed on 1.5% ethidium bromide agarose gels to confirm that single bands were produced.

The mRNA extracted was reverse transcribed by using High-Capacity cDNA Reverse Transcription Kit (Applied Biosystems, ThermoFisher Scientific), with starting mRNA levels at 1 μg for liver tissue and 0.5 μg for brain. Gene expression assays were performed in a CFX384 Touch™ Real-Time PCR Detection System (Bio-Rad, Hercules, CA, USA). Reactions used the SensiFAST™ SYBR^®^ No-ROX Kit (Bioline; Meridian Life Sciences, Memphis, TN, USA) with 3 mM Mg^2+^ and 0.8 mM dNTPs in 10 µL total reaction volumes. The 2-step cycling program began with 15 min at 95 °C, followed by 39 cycles at 10 s 95 °C and 20 s 60 °C, with data collected in real time. The reactions concluded with a melt curve analysis ranging from 65 °C to 95 °C in 0.5 °C increments at 5 s per step.

A minimum of two technical replicates were generated for each of eight samples per treatment group. From this, CFX Maestro^TM^ software (Bio-Rad, Hercules, CA, USA) calculated the cycle threshold (Ct) value, keeping Ct constant for each primer set. Comparative gene expression values (R-values) were generated according to the relative quantification of target gene transcripts in comparison to a reference gene transcript [88]. Accounting for primer pair efficiency (*E*) deviating from theoretical 100%, actual primer pair *E*-values were calculated using *E* = 10^(−/slope)^ −1 from the serially diluted standard cDNA estimate (reverse transcribed from RNA extracted from whole tadpole carcass). The variation in *E*-values was inspected to identify obvious systematic outliers, with *E* assumed to be the same per primer pair [89].

Gene expression was normalized to two reference genes, using the geometric mean of glyceraldehyde 3-phosphate dehydrogenase (*gapdh*) and ornithine decarboxylase (*odc*) [90]. Gene expressions were calculated as log_2_-transformed ratios [91,92] and shown relative to NEO-free controls (i.e., increased gene expression is >0, decreased gene expression is <0, and no change in gene expression is 0).

### 2.6. Sequencing

Representative qrtPCR products were purified using ExoSAP-IT PCR Product Cleanup Reagent (ThermoFisher Scientific) and sequenced using Sanger Sequencing. Sequence data were aligned and visually checked using SnapGene software (GSL Biotech, LLC; Chicago, IL, USA). Nucleic acid sequences of all genes and primers were aligned with ApE software, v. 2.0.49.10 (https://jorgensen.biology.utah.edu/wayned/ape/, accessed on 2 December 2021) [93], and the sequences were compared using NCBI-BLAST https://blast.ncbi.nlm.nih.gov/Blast.cgi, accessed on 2 December 2021 [94] to confirm amplification of the intended targets.

### 2.7. Liver Metabolism and Flow Cytometry

For measuring liver cell metabolic activity at U.S. Geological Survey (USGS) Wetland and Aquatic Research Center, tadpoles were mailed overnight in MBS and euthanized in a tricaine methane sulfonate solution for amphibian sedation [82]. After pooling five dissected livers per treatment group, individual cells were obtained by gentle mechanical homogenization as before. These cell suspensions were diluted with modified Simplified Amphibian Ringer’s solution (SAR; 116.0 mM NaCl, 1.4 mM CaCl_2_, 2.0 mM KCl, and 3.6 mM NaHCO_3_; 340 mosm kg^−1^, pH 7.2) [95] and filtered through 30 μm sterile nylon mesh (Component Supply, Sparta, TN, USA). Samples were maintained on ice until cell staining within 30 min and analyzed in triplicate. Control, metabolically inactive, dead cells were produced by a 10 min incubation in a 75 °C water bath. Cell suspensions were diluted to 10^6^ cells mL^−1^, then stained in triplicate in 200 μL aliquots. The staining controls consisted of either live or inactivated cells stained with SYTOX^®^ Green, C12-resazurin (cat. L34951; Invitrogen, ThermoFisher Scientific; LIVE/DEAD Cell Vitality Assay Kit), both stains, and unstained. The stains were prepared according to manufacturer’s instructions. Briefly, the resazurin stock was diluted to 50 μM in dimethylsulfoxide (DMSO), and the SYTOX working solution was 1 μM in DMSO. Stains were added to cells at 1 × 10^6^ mL^−1^ in SAR at pH 7.2 and 340 mOsm kg^−1^. Cells were incubated in the dark at 24 °C for 20 min prior to flow cytometric analysis.

Flow cytometry was performed with a FACScalibur (Becton Dickinson Immunocytometry Systems [BDIS], San Jose, CA, USA) with CellQuest Pro software v. 6.0 following calibration with FACSComp (BDIS), with fluorochrome excitation at 488 nm. Approximately 40,000 total cells per replicate were analyzed, with debris at the origin of a FSC (forward scatter) and SSC (side scatter) plot gated out. The primary threshold parameter for event collection was FSC, with FL-3H (fluorescence detection >670 nm) as the *x*-axis parameter and FL-1H as the y-axis (fluorescence detection at 515–545 nm). Two main subpopulations of live metabolic and injured/dead were gated, and the proportion of cells with reductase activity were determined by the gated event numbers. Additionally, the geometric mean fluorescence intensity of the injured/dead subpopulation was recorded. Data were analyzed with CellQuest (BDIS) and FlowJo software v. 10.6.1 (Tree Star, Ashland, OR, USA).

### 2.8. Statistical Analyses

Statistical analyses for differences among treatment groups following the first exposure period were performed by using JMP Pro version 13.1.0 [96], SigmaPlot version 13, and R Studio (Version 1.1.383) [97]. All tests were assessed at the significance level of α = 0.05, and standard errors (SE) were calculated for vivaria per treatment. Lack of independence among tadpoles was minimized by periodically moving the container locations and by intermittent photography to record body lengths with the randomly chosen tadpole subsets. Preliminary tests on normality and homogeneity of residuals were checked using Shapiro–Wilk and Brown–Forsythe tests, respectively. The incremental morphometrics during the first exposure period were analyzed by using one-way ANOVA with *post hoc* Tukey test [96], and a MANOVA comparison of time and treatment effect was applied to the length measurements [97]. A mixed (random) effect model using a nested analysis of tadpoles, vivarium, and treatment [96] also was applied to growth endpoints. The possibility of tadpole density effects was explored using the Holm multiple comparison test and Dunn’s non-parametric pairwise comparison (SigmaPlot) and EMS mixed (random) effects model (JMP). For both exposure periods (NF 47–57 and after NF 57 to tail resorption at NF 66), treatment effects on mortality and growth were assessed using a one-way ANOVA, Tukey’s, or *post hoc* Students *t*-test. Vivaria within treatment groups were replicates and tadpoles within a vivarium were considered a population. For the second exposure, a two-parameter logistic rate development model with a random effect (vivarium) and fixed effect (treatment) was analyzed by using SAS V9.4 [98]. This model was constrained so that no mean developmental stage would be greater than NF 66, allowing for variable initial developmental stages (day 44) and growth rates. For percent mortality at days 44 and 76, a two-way repeated measures ANOVA was performed; data were arcsine SQRT transformed [99]. Residuals were checked for normality (Shapiro–Wilk test) and homogeneity of variances (random scatter plot of residuals and fitted values). The repeated measures effect was significant as indicated by the null model likelihood ratio test (*p* < 0.0001) and included in the model; Tukey’s test was applied to compare means

Metabolic activity was analyzed using SAS 9.4 [98]. Residuals for all models were checked for normality (Shapiro–Wilk test) and homogeneity of variances (random scatter plot of residuals and fitted values). Nonparametric analyses were performed on ranked geometric means of the injured/dead population. Percentages of cells actively metabolic were arcsine (sqrt) transformed for a one-way ANOVA to test differences among treatment groups, with multiple comparisons performed with Tukey’s test. A nonparametric analysis on ranked geometric means was performed on injured/dead cell populations. Sampling events, experimental treatments and their interactions were analyzed for effects on liver cell metabolic activity and gene expression. For gene expressions, data were normalized for *odc* and *gapdh.* Student’s *t*-test was performed at *p* = 0.05 [96]. Figures and confidence interval representations were prepared [100].

All statistical analyses were performed at the level of significance of α = 0.05.

## 3. Results

### 3.1. NEO Concentrations

Analytical chemistry results obtained during exposure period 1 showed no significant difference between NEO concentrations in the culture media during the 24 h elapsed between daily solution changes (Supplementary Table S3). Thus, consistency of treatment exposures was verified.

### 3.2. Incremental Tadpole Lengths: Over the First Exposure Period

**Figure 1:**
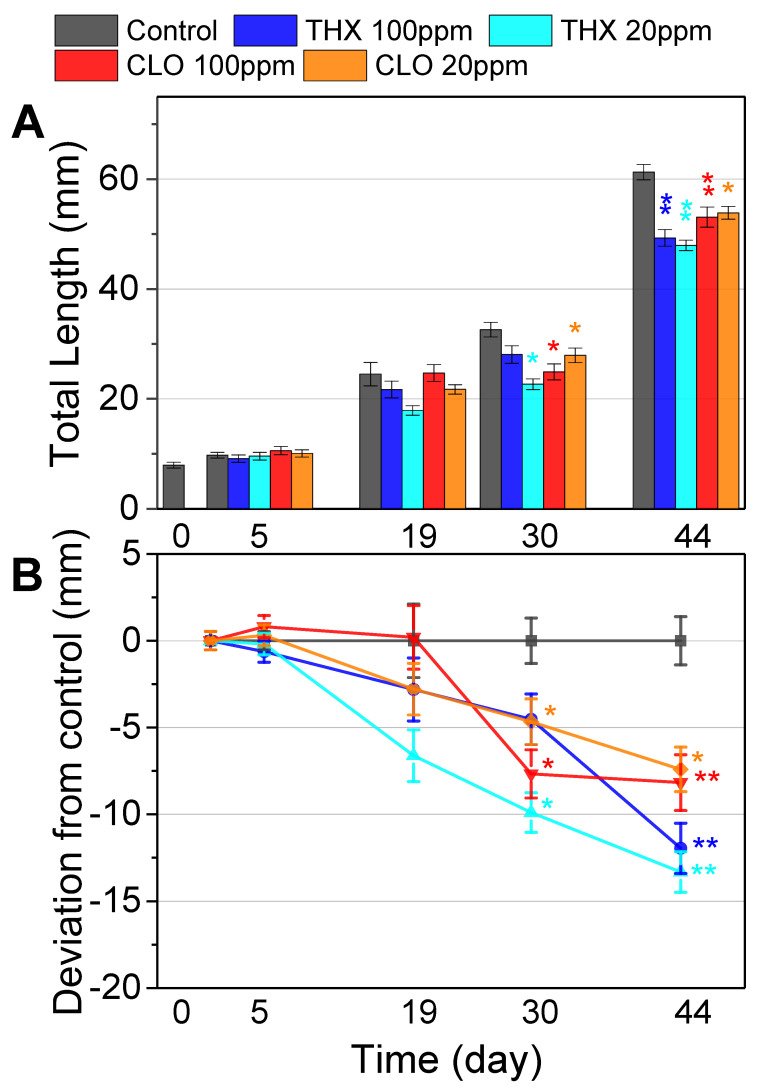
(**A**) Average total lengths (SE) of tadpoles (*Xenopus laevis*) measured incrementally from day 1 to day 44 post-hatch. Tadpoles were exposed in vivaria to thiamethoxam (THX), clothianidin (CLO), or pesticide-free control media. Lengths were measured from photographs of all individuals in each vivarium per treatment group (*n* = 4 replicate vivaria per treatment group). One asterisk indicates significant difference from control by ANOVA at the day of sampling, and two asterisks indicate significance by ANOVA and MANOVA (days 30 and 44). (**B**) The difference in tadpole lengths (SE) within treatment groups are plotted relative to control lengths.

On day 0, prior to allocation to the treatment groups, tadpole snout-to-vent lengths averaged 7.94 ± 0.53 mm). On days 5 and 19, no variation in length was noted among all treatment groups (Figure 1), yet on day 30, tadpole lengths in all treatments but THX high were significantly lower than that of the control. By days 30 and 44, reductions in total length were apparent in all treatments compared with the control (Figure 1); THX 100 ppm (*p* = 0.0265), THX 20 ppm (*p* < 0.0003), CLO 100 ppm (*p* < 0.0008), and CLO 20 ppm (*p* = 0.0093). Among all treatments, THX low induced the greatest reduction in total tadpole length over time (Figure 1; Supplementary Table S5).

### 3.3. Morphometrics: End of the First Exposure Period

**Figure 2:**
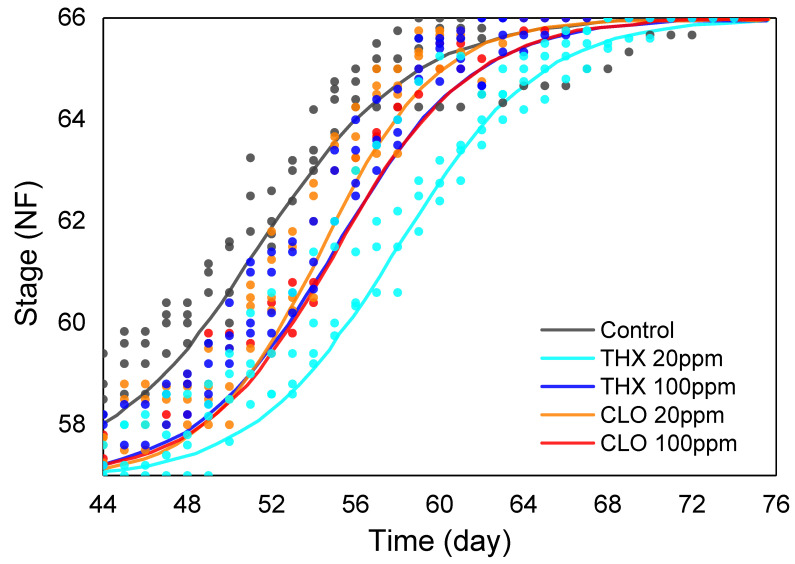
The progression of developmental stages of *Xenopus laevis* tadpoles during metamorphosis from the Nieuwkoop and Faber stages NF 57–66 in thiamethoxam (THX), clothianidin (CLO), or neonicotinoid-free control treatment groups. Each dot is the mean per one of four replicate vivaria. Solid lines were plotted after applying a two-parameter logistic growth model of developmental stage data from NF 57–66. The THX 20 ppm (low concentration) was depressed compared to that of the control group (*p* = 0.004).

By day 44 (NF 47–57), tadpoles in the control treatment group were heavier (0.73 g ± 0.11), longer (63.2 mm ± 2.50), and more developed (NF 56.9 ± 0.33) than the tadpoles in the other four treatments (Table 3; Figure 2). The THX low treatment most frequently reduced the morphometric measures pre-metamorphosis and was the only treatment impairing weight (Table 3). Compared to the controls, all four experimental treatments significantly retarded length (*p* ≤ 0.013) and developmental stage was significantly slowed (*p* ≤ 0.029), except the CLO high treatment (*p* = 0.054) (Table 3; Figure 2).

The random effects model attributed variance components of approximately 95% (96% for weight, 93% for length, and 95% for stage), and approximately 5% due to differences across vivaria (4% for weight, 7% for length, and 5% for stage). With one exception (THX high), variation among the four replicate vivaria per treatment at the end of the first exposure period was not significant, indicating no effects of density and a consistency of effect across the vivaria within each treatment group (0.105 < *p* < 0.934, Holm’s multiple comparison test; Supplementary Table S6). In THX high, tadpoles in one of the four replicate vivaria exhibited significantly smaller morphometric values than those in the other replicates (0.001 < *p* < 0.017, Supplementary Table S6). Further exploration of the significance observed within the THX high treatment revealed no relationship between difference in tadpole density and significance (Supplementary Table S6).

### 3.4. Developmental Stage: Over the Second Exposure Period

At the initiation of the second phase of NEO exposure at NF 57 (day 44), the developmental stage of the control treatment group was significantly more than all the other treatment groups (*p* < 0.001), ranging from 1.4 NF (THX high) to 2.1 NF (THX low) stages higher (Table 4). Over the next month, developmental stage of tadpoles in the THX low treatment group was depressed compared to that of the control group (*p* = 0.004) (Figure 2). Tadpoles in the THX high and each of the CLO treatment groups showed relatively similar developmental profiles, being slower than the control (Figure 2), albeit not significantly so (*p* = 0.06 to 0.16).

### 3.5. Mortality

**Figure 3:**
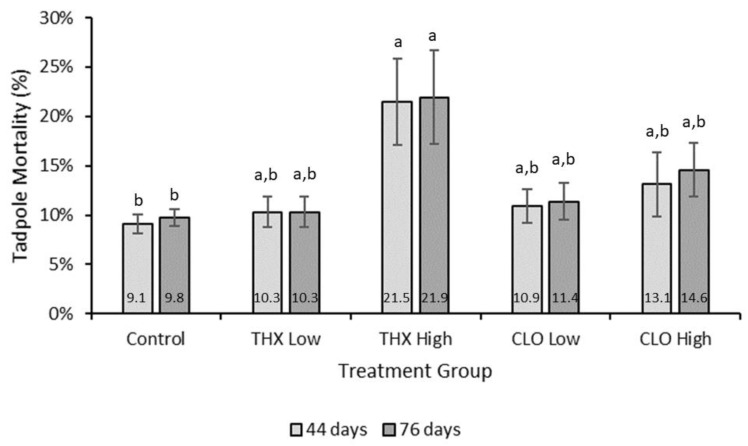
Cumulative mortality at days 44 and 76 of *Xenopus laevis* tadpoles exposed to thiamethoxam (THX) and clothianidin (CLO) treatments at high (100 ppm) and low (20 ppm) concentrations compared with that from neonicotinoid-free media. Differences among treatment groups at both days were noted, with control treatment mortality lower than THX high (*p* = 0.0007). Numbers within bars indicate the average mortality percentages. A different letter within a day group implies significance.

At the end of both exposure periods, cumulative tadpole mortality in the control treatment group was lower than THX high, at 9.1% (SE 0.9) and 21.5 % (SE 4.4) for day 44, and 9.8% (SE 0.8) and 21.9% (SE 4.8) for day 76 (*p* = 0.0007) (Figure 3). Mortality for both low treatments was similar at both time points, with THX low 10.3% (SE 1.5) and CLO low 10.9% (SE 1.7) on day 44 (Figure 3). Comparing cumulative mortality for both the time periods, up to metamorphosis then again to tail resorption among all treatment groups, mortality was different among treatment groups (*p* = 0.0007) with THX high ≥ CLO high, CLO low, THX low, >Control).

### 3.6. Liver Metabolism

**Figure 4:**
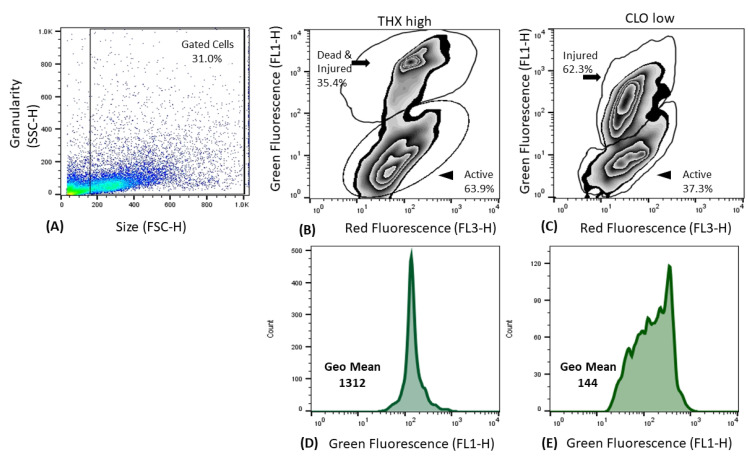
Representative flow cytometric cytograms from analysis of liver cells from 5 pooled organs (*n* = 3 replicates per treatment group) from *Xenopus laevis* tadpoles following exposure to neonicotinoids. (**A**) Debris is gated out at the origin, with 31.0% of intact, large cells further analyzed. (**B**,**C**) Reductases from cells from thiamethoxam 100 ppm (THX high) and clothianidin at 20 ppm (CLO low) indicate more metabolically active cells apparent in the THX high (63.9%) than the CLO low (37.3%), and higher levels of dead cells in THX high (**B**,**D**) with CLO lo showing a predominance of injured rather than dead cells (**C**). (**D**,**E**) Cells in the injured/dead gate are displayed in histogram format with the geometric means of green fluorescence (FL1-H) clearly showing higher values in THX high (1312) than in the CLO low treatment group (144). Data were collected 24 h after tadpoles were removed from the treatment.

**Figure 5:**
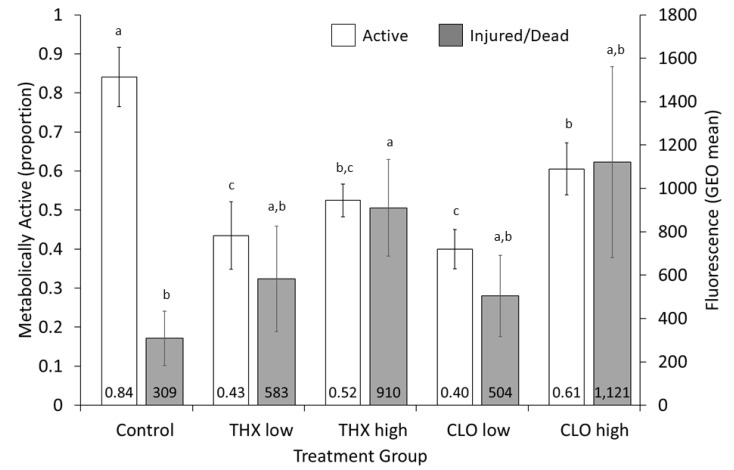
Flow cytometric data on the proportion of reductase activity and the cytotoxicity of the dead and injured cell populations from livers of *Xenopus laevis* tadpoles exposed for 44 days in neonicotinoid-free control media, or in either thiamethoxam (THX) or clothianidin (CLO) treatment groups at 20 and 100 ppm, or low and high, respectively. From 3 to 7 flow cytometric analyses were performed on 5 livers pooled per treatment (*n* = 24 total analyses). Data were collected 24 h after tadpoles were removed from the treatment and placed into neonicotinoid-free media. Letters represent differences among treatment groups per bar color (reductase activity, *p* < 0.0001; cytotoxicity, *p* = 0.0129).

Tadpoles of a similar age (day 44; NF 57) were analyzed at the end of the first exposure, after removal from their treatments for either 24 h (Figure 4 and Figure 5; Supplementary Figures S2 and S3) or 48 h (Supplementary Figures S2 and S3). For both endpoints—proportion of liver cells that were metabolically active (shown as red fluorescence; Figure 4) and the relative level of injured and dead cells as measured by geometric mean of green fluorescence by stain binding to nucleic acids inside damaged cell membranes (Figure 4)—no significant differences among treatment groups were noted when animals had been out of their treatment solutions for 48 h (Supplementary Figures S2 and S3). However, at 24 h significant differences in metabolic activity were noted among treatment groups (*p* < 0.0001), with controls showing the highest reductase level, and both low THX and CLO having the least (Control > CLO high ≥ THX high ≥ THX low = CLO low) (Figure 5, Supplementary Figure S2). At 24 h, the highest level of dead and injured cells was significantly different among treatment groups (*p* = 0.0129), with the most being shown with THX high and the least with control (THX high ≥ CLO high = THX low = CLO low ≥ Control, Figure 5, Supplementary Figure S3).

### 3.7. Gene Expression

**Figure 6:**
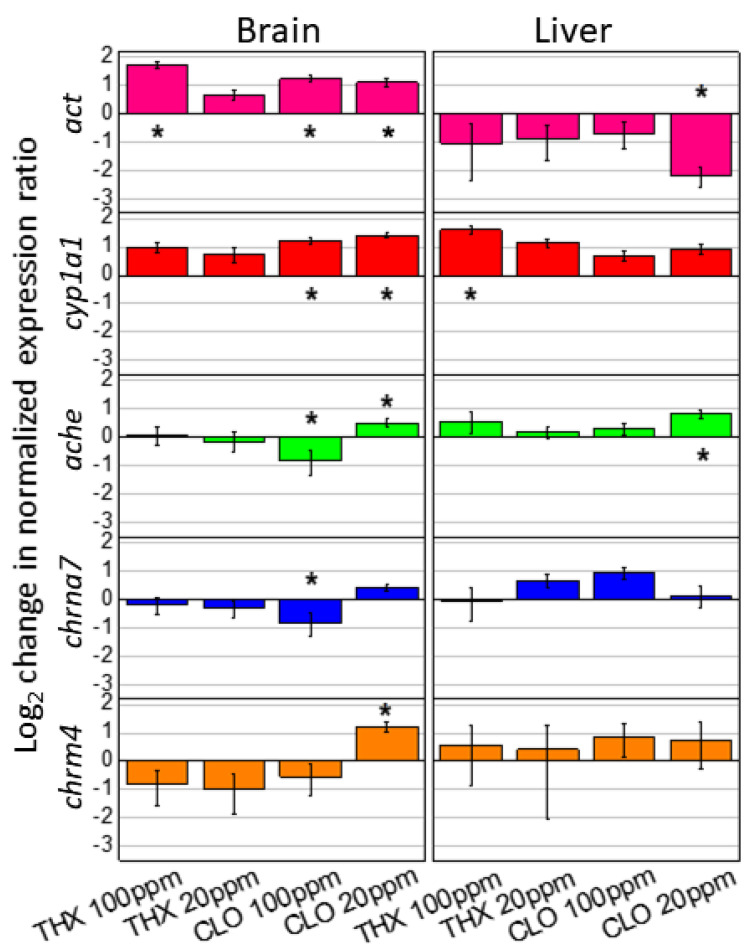
Gene expression levels of target genes muscarinic acetylcholine receptor 4 (*chrm4*), nicotinic acetylcholine receptor subunit *α*7 (*chrna7*), acetylcholinesterase (*ache*), cytochrome P450 (*cyp1a1*), and actin (*act*) in *Xenopus laevis* tadpole liver and brain tissues following exposure to thiamethoxam (THX) and clothianidin (CLO) at 20 and 100 ppm, or low and high, respectively, at 44 days. Target gene expressions were normalized to those from tadpoles in the control neonicotinoid-free treatment by using geometric means of both *odc* and *gapdh* reference genes; expression of *chrm4* was normalized to *odc* only. Asterisks denote values differing significantly from the pesticide-free treatment per tissue type.

In evaluating potential reference genes (Table 2), the expression of commonly used *act* and *ef1-a* varied across treatment groups, thereby necessitating their exclusion for use in the current study (Supplementary Figure S4). Conversely, expressions of both *odc* and *gapdh* were minimally variable across the treatment groups, thus they were used for expression ratios (Supplementary Figure S4). Then, because *act* expressions appeared to consistently increase or decrease depending on the tissue type, *act* was adopted as a target gene. In brain, *act* expression was consistently increased 3-fold more in the THX high and 2-fold more in both CLO low and CLO high treatment groups when compared to the control treatment (*p* = 0.0340) (Figure 6). Conversely in livers, *act* expression was decreased 5-fold in CLO low (*p* = 0.0196) (Figure 6).

More target gene expression differences over all treatments were demonstrated in brain (9/20 or 45%) than liver (3/20 or 15%), with CLO treatments accounting for 83% (10/12) of those expression differences observed over both tissue types (Figure 6). Expression of *cyp1a1* was upregulated in both brain (*p* = 0.026) and liver (*p* = 0.046). The increased expression ratio was highest in brain tissue exposed to CLO: at 20 ppm being 2.6-fold (*p* = 0.008) and at 100 ppm being 2.3-fold (*p* = 0.024). Increased liver *cyp1a1* expression was seen in THX treatments, up to 3.7-fold higher, however only significant for THX 100 ppm (*p* = 0.020). The only change in gene expression related to the NEO-target receptor protein subunit (*chrna7*; nicotinic cholinergic receptor α7) was demonstrated in brain, being reduced within treatment group CLO high (*p* = 0.022), but no changes noted were from liver (Figure 6). Similarly, with *chrm4* expression (relevant to THX binding), no significant changes were noted among treatment groups in liver, but an increase was noted with CLO low (*p* = 0.037) in brain. In brain, acetylcholinesterase (*ache)* expression was reduced by CLO high (*p* = 0.031) but increased by CLO low (*p* = 0.038; Figure 6). In liver, *ache* was increased with CLO low (*p* = 0.016; Figure 6).

## 4. Discussion

Amphibians breed and grow in wetlands and ephemeral ponds, thus are considered sentinel species in aquatic habitats since crucial phases of their development occur in water, and they generally do not venture far from where they were hatched. Larval stages, in particular, are model vertebrate bioindicators due to their sensitivity to environmental stressors and exposure risk [101]. Although NEO are used extensively in agriculture and occur in nearby aquatic ecosystems, only a limited number of studies with amphibians have been performed to discern potential biological effects. Most of the response variable data reported include survival, growth, development, and behavior. Recently, there has been interest in blood profiles and corticosterone as reflecting immune competence, whereby more integral ecotoxicological studies have been encouraged, with some relevant to disease occurrence and parasite infestation [30,40,102,103,104,105].

### 4.1. Survival

In this study, at the end of both exposure periods, NF 57 (day 44) and NF 66 (day 76), the cumulative tadpole mortality in the THX high treatment group at 21.5% was significantly higher than that in the control treatment group (9.1%, *p* = 0.0007; Figure 3). Because THX is converted to CLO, as its principal metabolite, CLO may have contributed to THX toxicity [19]. Although mortality in CLO high (13.1%) was slightly higher than that in THX low and CLO low (10.3% and 10.9%, respectively), these values were not significantly different from control or THX high (Figure 3).

In previous controlled studies with other juvenile amphibian species exposed to CLO or THX, no mortalities occurred (Table 5); the studies differed in exposure times and chemical formulations, and the concentrations used were representative of concentrations measured in surface waters [105,106]. The previous studies’ lack of mortalities is in contrast with the results in this study, likely due to the a.i. concentrations used herein. In addition to addressing the risk assessment needs of USEPA for CLO and THX with amphibians, the results underscore the importance of THX metabolite contribution to both survival and sublethal effects in this aquatic larval model amphibian.

In this study, the a.i. in the THX and CLO commercial formulations were 23% and 77%, respectively, and the a.i. of the eight analytically tested water samples from the vivaria were within an average of 5% of the target a.i. concentrations (Supplementary Table S3). Possible effects of the “inert” ingredients of commercial formulations cannot be discounted. Such exposures could complicate interpretations of results. For example, in a 24 h exposure to a commercial formulation of imidacloprid (IMI) with the a.i. concentration well below the published LC_50_ concentrations, wood frog (*Lithobates (Rana) sylvatica*) tadpoles suffered unexpectedly high mortality rates [103]. For this study it is worth noting that the sublethal biomarker data were collected from survivors with apparent higher fitness or less sensitivity than the mortalities may have displayed.

### 4.2. Growth and Development

Biological phenomena, with fitness traits such as survival, growth, and reproduction, as well as metabolism and immune responses, can display non-linear dose response relationships, or hormetic responses. Hormesis is a dose-response phenomenon characterized by low-dose stimulation and high-dose inhibition, with the mechanism reliant on the same agonist interacting with different receptor subtypes [107]. In the current study with *Xenopus* tadpoles, morphometric responses in the THX low treatment were counterintuitive: greatest reduction in length over time (Figure 1; Supplementary Table S5), the only treatment that impaired weight at NF 47–57 (Table 3), and the only one to depress developmental stage compared to control during the second exposure period (Figure 2). Conversely, by the end of the first exposure period, developmental stage was significantly slowed in all treatments, except the CLO high treatment (Table 3). A 21-day exposure of adult Northern leopard frogs (*Lithobates pipiens*) to the NEO IMI, showed that the control group lost the most body mass and the group exposed to 500 ppm IMI lost the least body mass, indicative of a hormetic growth response [108]. When Northern leopard frog tadpoles were exposed to CLO, changes in leukocyte profiles occurred only at the lower concentrations tested, with no changes in survival, lengths, or time to development at any of the concentrations tested [101]. Unimpaired growth can be a fitness advantage (Figure 1; Supplementary Table S5). Interestingly, larval development was advanced in Northwestern salamanders (*Ambystoma gracile*) exposed to IMI after 35 d, indicating thyroid endocrine axis interference [109]. In this study, variable results with morphometrics and development suggest endocrine effects (Table 3 and Table 4, Figure 1 and Figure 2, Supplementary Table S7). Together these studies underscore complexities in biomarker responses and NEO chemistry, necessitating diligence in conducting experiments that consider dose, life stage, and biological endpoints at multiple levels of biological organization along the AOP.

Multiple reasons, such as differences in site locations in field studies, amphibian life stages, variabilities in receptor protein constructs, and exposure times could account for the discrepancy in loss of survival observed at concentrations thousands of times lower than the published values. These factors underscore the necessity of analytical testing for a.i., especially in non-traditional animal exposures.

### 4.3. Liver Cell Function

Studies on metabolism of commercial NEO are inherent to the EPA registration process for approved uses [19]. Chemical reactions here involve initial oxidation or reduction as both activation and detoxification mechanisms; the metabolic alteration to CLO involving N-demethylation, and THX converted to CLO by ring methylene hydroxylation [19]. The cytochrome P450 enzyme superfamily of heme enzymes includes thousands of isoenzymes identified in multiple biological kingdoms [110]. Assays detecting P450-dependent enzymatic activity involved in detoxification processes (e.g., EROD activity; 7-ethoxyresorufin-O-deethylase activity) in response to pollution events have been used for over two decades as a proven biomarker in investigations of contaminant exposures [111]. The *cyp1a* enzyme converts 7-ethoxyresorufin into measurable fluorescent resorufin. The flow cytometric assay used here is based on the reduction of C12-resazurin to red fluorescent C12-resorufin in metabolically active cells, as it enters living cells in its non-fluorescent form and is reduced by oxidoreductases in mitochondria, microsomes, and the cytosol to the fluorescent product, C12-resorufin. The uptake of the cell-impermeant green fluorescent nucleic acid stain, SYTOX Green occurs in cells with compromised plasma membranes, typically late apoptotic and necrotic cells. The assay is powerful, in that multiple distinct subpopulations reflect both metabolic activity and concurrent levels of injury. For instance, a sample of pooled livers from THX high showed more cells (63.9%) metabolically active than a sample from CLO low (37.3%), yet fewer dead and injured cells were apparent in THX high (35.4%) than in CLO low (62.3%) even with the level of dead and injured cells in THX being much higher than in CLO low as measured by GEO means (1312 versus 144, Figure 4).

Amphibian decontamination mechanisms generally display lower liver metabolic activity than those of mammals [112]. Factors that may influence results include species, reproductive stage, age, and amphibian life stages [111,113]. Thus, for this study, after 24 h of being removed from treatments, liver metabolic activity in tadpoles at a similar age (NF 57; *p* < 0.0001) was different among treatments (Figure 5, Supplementary Figure S2). These measurable enzyme level differences at 24 h reflect induction of decontamination mechanisms, a clear biomarker of exposure. Yet, because no differences among treatment groups were noted in metabolism or cytotoxicity after tadpoles had been removed for 48 h (Supplementary Figures S2 and S3), these results indicate metabolism actuating over time. Conversely, cytotoxicity at 24 h measured by GEO mean was lowest in control, being significantly different than the most cytotoxic in THX high (Figure 5; *p* = 0.0129). The results for *Xenopus* tadpoles indicate comparatively more metabolically active cells in the control and high CLO treatments than in the THX high, THX low, and CLO low. For the dead and injured cell subpopulations, the higher mean fluorescence index shown by THX high treatment reflects higher cytotoxicity than low treatment of both THX and CLO. These results may be influenced by the metabolic conversion of THX to CLO, which may help to explain mortality results and account for why THX is more injurious at these concentrations than CLO. Generally, these results indicated that the higher concentrations of the THX and CLO are more cytotoxic and induced less injury to liver cells than do lower concentrations (Figure 5).

### 4.4. Gene Expression

Cholinergic systems of many non-neural tissues signal locally [45,114], with a family of nAChRs being differentially expressed in numerous tissues. In a study with Northern leopard frogs, IMI and the IMI-metabolite were detected in brains, indicating the ability to cross the blood brain barrier [108], suggesting the utility of testing other NEO. In each of the tissues from the exposed freshwater cichlid *Australoheros facetus*, including liver, brain, muscle, and blood, IMI was detected [115]. Generally, however, nervous system tissues have comparatively low amounts of biotransformational metabolic processes, operating primarily in the liver, with microsomal CYP450′s encoded by cytochrome P450 genes [58,110]. Metabolism of the specific NEO either increases or decreases potency depending on the compound and specificity of the nAChR [58]. In this study, brains and livers were selected for transcript detections to measure expression levels for NEO receptor subunits (*chrna7* for α7-nAChR protein; *chrm4* for subunit 4 of mAChR), cell structure (*act*), cell function (e.g., cell signaling or neurotransmission; *ache*), and decontamination processes (*cyp1a1*).

#### 4.4.1. Reference Genes and *Cyp*

In the assessment of the reference genes considered for use in this study (*act*, *efl-a*, *gapdh*, and *odc*), results indicated that only *odc* and *gapdh* were suitable for normalizing potential gene expression changes in tissues from tadpoles exposed to the NEO treatments, suggesting their further candidacy in additional NEO studies with amphibians. Actin is multifunctional, forms microfilaments, and facilitates a multitude of protein-protein interactions involving regulation of transcription, cell shape, and motility [116]. Frequently employed in studies as a house-keeping gene, *act* was differentially expressed here in brain and liver, being up-regulated in brain in all treatments except THX low, yet down-regulated in liver, and significantly so with CLO low (Figure 6). Because metamorphosis is a critical time of transition when metamorphic hormones orchestrate the loss or reorganization of many tissues and organ systems [37], the fluctuations in *act* expressions observed at this life stage might not be unexpected and are likely not as pronounced at later amphibian life stages.

Other studies bolster the futility of using *act* with juvenile *X. laevis*. After analytical grade IMI exposure to a sensory neuronal cell line (F11 cells—an immortalized cell line derived from a fusion between mouse neuroblastoma and rat ganglia), changes in cytoskeletal structure were detected with immunofluorescent microscopy after binding by anti-actin antibodies [117]. Additionally, in an exposure study with the OP insecticide, chlorpyrifos, subsequent AChE activity and muscular damage in *X. laevis* juveniles were most prevalent with concentrations that most inhibited AChE, crucial for neurotransmission [118]. In this study, CLO low induced both *act* and *ache* expression levels that were different than those in both control brain and liver (Figure 6). In brain, three of the four treatments resulted in increased *act* expression, yet in liver, *act* expression was reduced in each treatment group, with CLO low showing a significant reduction (*p* < 0.05, Figure 6). Additionally, expression of *ef-1-*α fluctuated within treatment groups, although to a lesser extent than *act*, resulting in its exclusion as a reference. This gene encodes a highly conserved protein involved with translation in protein synthesis [119]. Although *act* and *ef-1-α* often are used as default reference genes in transcription experiments, these results suggest a cautionary approach, especially in studies during dynamic amphibian life stages. By carefully comparing the expression of the four reference genes tested in this study, the calculated expression ratios were assured by excluding those that fluctuated among treatment groups, while maintaining the use of two (*gadph* and *odc*, Table 2, Figure 6). Moreover, these results with *act* expression inform interpretations of the tadpole reductions in growth and development noted at the end of the first exposure period (Table 3 and Table 4, Figure 1 and Figure 2, Supplementary Table S7).

In this study, *cyp1a1* expressions trended to increase across the treatment groups for both brain and liver, but only significantly with both CLO treatment groups in brain and THX high in liver (Figure 6). This upregulation suggests a broad detoxification response in *X. laevis*. In the brains of tadpoles of the bullfrog (*Rana catesbeianus*) following exposure to 17 α-ethynylestradiol and atrazine, an increase of P450 aromatase (*CYP19*) expression was noted [120]. The action of P450 enzymes, having a broad substrate specificity, results in either bioactivation or detoxification, with enhanced detoxification processes concurrent with insecticide resistance development [121].

Non-target amphibians can develop resistance to pesticides, with populations displaying cross-tolerance among pesticides that share, or differ, in modes of action [76]. Short-term exposure to carbaryl altered population dynamics under field conditions, with survival to metamorphosis of Woodhouse’s toad (*Bufo woodhousii*) nearly doubled, but not for Southern leopard frogs (*Rana sphenocephala*) [39]. Multiple exposures of carbaryl in outdoor cattle tanks were shown to stimulate metamorphosis in green frog (*Rana clamitans*), even more than single exposures [8], and wood frog (*Lithobates sylvaticus*) populations located closer to agricultural land use were more tolerant to chlorpyrifos [122]. Thus, a short-lived contaminant may alter population dynamics and endpoints such as metamorphosis many days after exposure [39]. With studies performed at the cell and molecular levels (e.g., *cyp1a1* expression and liver metabolism) in amphibians, during the biological phases when NEO first interrelates with the animal, the later non-target amphibian endpoints (e.g., mortality, development, and growth) can be better understood.

#### 4.4.2. Receptors and Acetylcholinesterase

AChE catalyzes the hydrolysis of acetylcholine into choline and acetic acid at the synaptic junctions, facilitating nerve impulse transmission. As a common biomarker for OP and other contaminants, AChE has served as an indicator of neurological toxicity in both vertebrates and invertebrates [18]. Binding of OP inactivates these enzymes, and normal nervous system function is disrupted as acetylcholine accumulates at the synapse [50]. In order to reverse the binding, more AChE is synthesized [50]. By the NEO blockage of acetylcholine binding, AChE accumulates causing neurologic toxicity [10]. In this study, because CLO low reduced *ache* expression and CLO high increased it, neurotransmission and cell signaling may have been differentially influenced by CLO concentration, potentially with inhibition or induction of AChE activity, respectively (Figure 6). The presynaptic mAChRs are involved with both the inhibition and enhancement of acetylcholine release, with both mAChRs and nAChRs widely distributed in the central nervous system [123]. Both receptor types are involved in cognition and locomotor activity, with NEO-induced neurobehavioral and biochemical adverse effects related to chemical structure and receptor subunit interaction [52].

This study with *X. laevis* indicated CLO low induced gene expression changes more frequently (15%) than any of the other treatments, with either CLO low or CLO high sometimes inducing different results (e.g., CLO low increased *ache* and *chrm4* expressions and CLO high reduced *ache* and *chrna7* expressions). Of the 40 calculated expression ratios in brain and liver, 25% were detected from tadpoles in CLO treatment groups (most frequently in brain; 22.5% of the incidences). Low CLO treatment increased *ache* gene expression in both brain and liver, whereas high CLO reduced expression in brain (Figure 6). For both receptor types in this study, expression changes occurred in brain only, with CLO high reducing expression of *chrna7* (codes for α7-nAChR protein) and CLO low increasing *chrm4* expression (codes for subunit 4-mAChR) (Figure 6). In rats, CLO was found to activate nAChR subunits, including α7 and the mAChRs, inducing dopamine release from striatum [52]. In *Xenopus* oocytes expressing nAChR, its distribution and role in neurotransmission was demonstrated by receptor binding and blockage with bungarotoxin [124].

## 5. Summary

The connection to environmental pollutants throughout their life cycle and their physiology makes amphibians good bioindicators for conditions in the environment, and a recent model of interest for studies with NEO. The NEO are water soluble and persistent, having a half-life in soil that varies among compounds and across studies [21]. Thus, with repeated application and runoff and due to unforeseen hydrologic and atmospheric processes, concentrations of NEO in waters under particular conditions could reach relatively high levels [16,67,74,75] such as those used in this study. Using CLO and THX at the high of 100 ppm and low at 20 ppm, mortality of *X. laevis* tadpoles was 21.5% when exposed from NF 47-NF 57 to THX high, as compared with the treatment group at 9.1%. If sublethal effects can be detected at approximately 1/10th the LC_50_ [22], this may be a starting point for exploring LC_50_ with this species and THX.

The AOP for THX and CLO in *Xenopus* tadpoles linked the molecular and cellular initiating events to outcomes relevant to assessing risk for aquatic phase amphibians. Direct and indirect effects of NEO on subtle sublethal endpoints, and the influence of multiple interacting stressors at various life stages, however, is needed to fully understand the effects of NEO on amphibians. Developmental stage was significantly slowed in all treatments, except CLO 100 ppm. The THX 20 ppm most significantly reduced the morphometric measures pre-metamorphosis, being the only treatment to impair weight gain. The higher levels of THX and CLO were more cytotoxic and induced less injury to liver cells than did the lower NEO levels. When selecting reference genes for amphibians undergoing physiological changes in the metamorphic life stages, *act* was found to be useful as reflective of potential cell structure alterations. Gene expression changes related to nAChR and mAChR were indicated, especially in brain. This study established new linkages between the THX and CLO binding receptors in exposed to *X. laevis* tadpoles, with measurable responses in organism growth, survival, cell metabolism, and gene expression levels.

## Figures and Tables

**Table 1:** Compilation of results on fish organismal response after exposure to clothianidin or thiamethoxam.

Common Name	Scientific Name	Biological Endpoint	Media Type	Exposure Concentration of the Active Ingredient (mg L^−1^)	Observation Duration (Days)	Data Source
Clothianidin						
Bluegill sunfish	*Lepomis macrochirus*	LC_50_ ^1^; mortality	freshwater	>117	4	EPA #344, 1992
Bluegill sunfish	*Lepomis macrochirus*	NOEL ^2^; mortality	freshwater	117	4	EPA #344, 1992
Fathead minnow	*Pimephales promelas*	LOEC ^3^; growth	n.d.	20	33	EPA #344, 1992
Fathead minnow	*Pimephales promelas*	NOEL; growth	n.d.	9.7	33	EPA #344, 1992
Fathead minnow	*Pimephales promelas*	EC_50_; intoxication	freshwater	>0.5	4	DePerre et al., 2015
Fathead minnow	*Pimephales promelas*	LC_50_; mortality	freshwater	>0.5	4	DePerre et al., 2015
Sheepshead minnow	*Cyprinodon variegatus*	NOEL; mortality	saltwater	93.6	4	EPA #344, 1992
Rainbow trout	*Oncorhynchus mykiss*	LC_50_; mortality	freshwater	>105.8	4	EPA #344, 1992
Rainbow trout	*Oncorhynchus mykiss*	NOEL; mortality	freshwater	105.8	4	EPA #344, 1992
Zebrafish	*Danio rerio*	multiple	freshwater	20.0	5	Padilla et al. 2012
Thiamethoxam						
Bluegill sunfish	*Lepomis macrochirus*	LC_50_; mortality	freshwater	114	4	EPA #344, 1992
Bluegill sunfish	*Lepomis macrochirus*	NOEL; mortality	freshwater	114	4	EPA #344, 1992
Sheepshead minnow	*Cyprinodon variegatus*	LC_50_; mortality	salt water	>111	4	EPA #344, 1992
Sheepshead minnow	*Cyprinodon variegatus*	NOEL; mortality	salt water	111	4	EPA #344, 1992
Rainbow trout	*Oncorhynchus mykiss*	LC_50_; mortality	fresh water	100	4	EPA #344, 1992
Rainbow trout	*Oncorhynchus mykiss*	NOEL; mortality	fresh water	100	4	EPA #344, 1992
Rainbow trout	*Oncorhynchus mykiss*	LC_50_; mortality	n.d.	>1005	4	Syngenta SDS
Rainbow trout	*Oncorhynchus mykiss*	LC_50_; mortality	fresh water	>100	4	EPA #344, 1992
Rainbow trout	*Oncorhynchus mykiss*	NOEC ^4^	fresh water	20	45	EPA #344, 1992
Rainbow trout	*Oncorhynchus mykiss*	LOEC	n.d.	>20	45	EPA #344, 1992

^1^ LC(50) = 50% lethal concentration in treatment. ^2^ NOEL = No observable effect level, or the highest exposure level of a substance or material that produces no noticeable toxic effect. ^3^ LOEC = lowest observed effect concentration different from control. ^4^ NOEC = No observed effect concentration or highest dose level where no effects are noted.

**Table 2:** Target and reference genes, their general cellular function, primer pair sequences ^a^, and size in base pairs.

Gene (Abbreviation)	Cellular Function	Sequence 5′-3′	Base Pairs
Reference Genes			
Elongation factor 1-α (*ef1-a*) ^b^	Delivery of aminoacyl tRNAs to ribosomes	CTG CAC ATA TCG CCT GTA AG	107
		GGC AGC ATC TCC AGA TTT C	
Actin (*act*) ^b^	Major protein of contractile apparatus	GGC CGT ACA ACT GGT ATT G	93
		CAT GAT GGC ATG AGG TAA GG	
Ornithine decarboxylase (*odc*) ^b^	Polyamine biosynthesis pathway	GTA CAA GCT GTC TCA GAT GC	92
		GGG AAT CCA CCA CCA ATA TC	
Glyceraldehyde-3-phosphate dehydrogenase (*gapdh*) ^b^	Carbohydrate metabolism	ATC AAG GCC GCC ATT AAG	115
		CAA AGA TGG AGG AGT GAG TG	
Target Genes			
nAcetylcholine receptor subunit α7 (*chrna7*) ^c^	Mediating synaptic fast signal transmission	ACC TGA AGT TTG GCT CAT GG	159
		GGT ATG GTT CCT TGC AGC AT	
Muscarinic acetylcholine receptor 4 (*chrm4*) ^c^	Mediating synaptic fast signal transmission	ATC TTT ATC GCC ACC GTC AC	204
		GAG TGG CCA GTA ACC CTT GA	
Cytochrome p450 (*cyp1a1*) ^c^	Monooxygenase; detoxification, resistance	AGG AGA AGA GAG TCG ATG	220
		GCT CTG TCT GAT AAT CTA GG	
Acetylcholinesterase (*ache*) ^c^	Hydrolyses acetylcholine, ends CNS signal	ATC TGA ACT ATA ACC CAC AG	245
		TGT AAT GTT GAG CAG TTT AG	

^a^ Sequence data and descriptions obtained from RefSeq (https://www.ncbi.nlm.nih.gov/refseq/, accessed on 2 December 2021), GenBank (Benson et al., 2009) [84], and XenBase (Karimi et al., 2019) [60]. ^b^ Source was per Dickinson and Sive, 2006 [87]. ^c^ Primers designed for this study.

**Table 3:** Morphometrics (SE) of *Xenopus laevis* tadpoles at the end of 44 days of exposure to thiamethoxam (THX), clothianidin (CLO), or in neonicotinoid-free media in four replicate vivaria per treatment.

Treatment	Tadpoles (*n*) ^a^	Wet Weight (g)	*p* Value	Total Length (mm)	*p* Value	NF Stage	*p* Value
Control	45.8 (4.3)	0.73 (0.11)	-	63.18 (2.50)	-	56.9 (0.33)	-
THX 20 ppm	69.5 (3.2)	0.39 (0.03)	**0.022 ^b^**	48.22 (1.22)	**<0.001**	54.2 (0.18)	**0.001**
THX 100 ppm	68.3 (3.9)	0.52 (0.07)	0.204	52.12 (2.91)	**0.006**	54.5 (0.59)	**0.002**
CLO 20 ppm	52.0 (3.4)	0.56 (0.04)	0.245	54.00 (1.56)	**0.013**	55.3 (0.43)	**0.029**
CLO 100 ppm	44.8 (4.1)	0.62 (0.10)	0.334	53.79 (1.86)	**0.007**	55.7 (0.35)	0.054

^a^ Average tadpole number in the four replicate vivaria per treatment group. ^b^ Bold is significant by ANOVA with Holm-Sidak multiple comparison method, α = 0.05, comparing treatments with control.

**Table 4:** Comparison of *Xenopus laevis* tadpole development ^a^ in treatment groups compared with controls in neonicotinoid-free media as measured on day 44 and on day 76. Tadpoles were exposed to low or high concentrations of clothianidin (CLO) and thiamethoxam (THX).

Treatment	Difference ^b^ in NF (95% Confidence Interval)	
	NF 57; day 44	Post-metamorphosis (NF 66; day 76)
CLO 20 ppm	**1.8 (1.1, 2.4) ^c^**	**1.33 (1.16, 1.50)**
CLO 100 ppm	**1.5 (0.7, 2.3)**	1.16 (0.97, 1.34)
THX 20 ppm	**2.1 (1.5, 2.7)**	1.08 (0.96, 1.20)
THX 100 ppm	**1.4 (0.8, 1.9)**	1.12 (0.99, 1.25)

^a^ Developmental staging by Nieuwkoop and Faber (NF) (Gurdon, 1995). ^b^ Difference is defined as the NF of the control minus the NF of the experimental treatment value. ^c^ Bold indicates significance at *p* < 0.001.

**Table 5:** Compilation of results on amphibian survival after exposure to clothianidin or thiamethoxam ^1^.

Common Name	Scientific Name	Life Stage	Exposure Concentration (ug/L)	Duration (Days)	Mortality	Data Source
Clothianidin						
Northern leopard frogs	*Rana pipiens*	tadpole	0–100	56	no	Robinson et al., 2021
Northern leopard frogs	*Rana pipiens*	tadpole	428 ± 66	14	no	Gavel et al., 2021
Northern leopard frogs	*Rana pipiens*	tadpole	2.5 and 250	on order	no	Robinson et al., 2019
Wood frogs	*Lithobates sylvaticus*	tadpole	2.5 and 251	on order	no	Robinson et al., 2020
Thiamethoxam						
Northern leopard frogs	*Rana pipiens*	tadpole	0–100	56	no	Robinson et al., 2021
Northern leopard frogs	*Rana pipiens*	tadpole	304 ± 49	14	no	Gavel et al., 2021
Northern leopard frogs	*Rana pipiens*	tadpole	2.5 and 250	on order	no	Robinson et al., 2019
Wood frogs	*Lithobates sylvaticus*	tadpole	2.5 and 251	on order	no	Robinson et al., 2020
Wood frogs	*Lithobates sylvaticus*	tadpole	1, 10, 100	42	no	Robinson et al., 2017
Wood frogs	*Lithobates sylvaticus*	tadpole	200–25,200	14	n.a.	Pochini and Hoverman, 2017

^1^ Commercial formulations may have been employed.

## Data Availability

Data from this study are available in two USGS data releases: Jenkins, J.A.; Brown, B.L. *Xenopus* metamorphosis after neonicotinoid exposure. U.S. Geological Survey data release 2021, https://doi.org/10.5066/P97PROVJ; Jenkins, J.A.; Brown, B.L. Gene expression and liver cell metabolism from *Xenopus laevis* tadpoles exposed to neonicotinoids. U.S. Geological Survey data release 2021, https://doi.org/10.5066/P9KW3G2G.

## Supplementary Materials

The following are available online at https://www.mdpi.com/article/10.3390/ijms222413291/s1.
