# Supplementary material for: Juvenile African Clawed Frogs (Xenopus laevis) Express Growth, Metamorphosis, Mortality, Gene Expression, and Metabolic Changes When Exposed to Thiamethoxam and Clothianidin
Source: Int J Mol Sci. 2021 Dec 10;22(24):13291. doi: 10.3390/ijms222413291 (PMC8706403; doi:10.3390/ijms222413291)
Supplement: Supplementary file 1 [file ijms-22-13291-s001.zip › ijms-1406152-SI.pdf]

Supplementary Information

Supplementary Table S1

S Table S1. Tadpole numbers per treatment group at the start of the first and second exposure periods[a] to thiamethoxam (THX) or clothianidin (CLO).

Treatment (ppm)	Vivarium	Exposure 1	Exposure 2
Pesticide-free media	C1	38	5
(0 ppm)	C2	39	5
	C3	51	4
	C4	55	5
THX (20 ppm)	TL1	62	5
	TL2	77	5
	TL3	67	4
	TL4	72	5
THX (100 ppm)	TH1	74	4
	TH2	57	5
	TH3	73	5
	TH4	69	5
CLO (20 ppm)	CL1	51	4
	CL2	43	4
	CL3	55	4
	CL4	59	4
CLO (100 ppm)	CH1	51	4
	CH2	41	3
	CH3	52	-
	CH4	35	-
	Total tadpoles	1121	80

The first exposure was from tadpole days 1-44 which spanned stages NF 47-57. The second exposure was days 45-76 which spanned NF 57-66.

Supplementary Table S2

S Table S2. Tadpole feed for juveniles.

	Components and Instructions	Amount
1	DI Water	0.25 L
2	Organic nettle powder	2.5 g
	Mix #1 and #2, boil, then cool	
3	DI Water	0.75 L
4	Fish flakes, generic	2.5 g
5	Nasco[1] Frog Brittle powder (SB09480LM,M)	12.5 g
	Blend or homogenize 1-5, aliquot, and store at 4°C for up to 1 week.	

[1]Nasco, Fort Atkinson, WI.

At Day 1, each vivarium contained approximately 50 tadpoles (n=56±13; total 1121 tadpoles). The average numbers of tadpoles in each treatment group over the first exposure period ranged from 44.8 ± 4.1 to 69.5 ± 3.2. Any sorption of the NEO to feed was kept consistent by performing the first feeding after the daily media change, and the second 6-7 hours after the first feeding.

Supplementary Table S3

S Table S3. Measured concentrations[1] of clothianidin (CLO) and thiamethoxam (THX) from *Xenopus laevis* tadpole exposure vivaria. Samples were collected immediately at the beginning of a treatment exposure (initial) and after 24 hours just prior to the next treatment replacement. Treatments were refreshed daily.

Treatment	Concentration (ppm)	Timing	Measured Concentration (ppm)
CLO	20	Initial	22.3
	20	Final	23.5
	100	Initial	98.6
	100	Final	97.6
THX	20	Initial	19.6
	20	Final	20.8
	100	Initial	96.0
	100	Final	96.6
Control	0	.	n.d.

[1] Analysis was performed by liquid chromatography tandem mass spectrometry (Hladik and Calhoun, 2012) [79].

Supplementary Table S4

S Table S4. Thiamethoxam and clothianidin agricultural formulations used in this study.

Neonicotinoid	CAS[1]	Commercial Name	Company	Active ingredient (%)	Lot no.	$LogK_{ow}$ at 25°C[2]
Thiamethoxam	153719-23-4	Platinum 75 SG Insecticide	Syngenta Crop Protections, LLC., Greensboro, NC	75.0	322029	-0.13
Clothianidin	210880-92-5	Belay	Valent, USA Corporation, Walnut Creek, CA	22.3-23.7	17420324163Y	0.91

[1]Chemical Abstracts Service Registry numbers, which is a registered trademark of the American Chemical Society.

[2]The Log K_{ow} is the octanol-water partitial coefficient.

Supplementary Figure S1

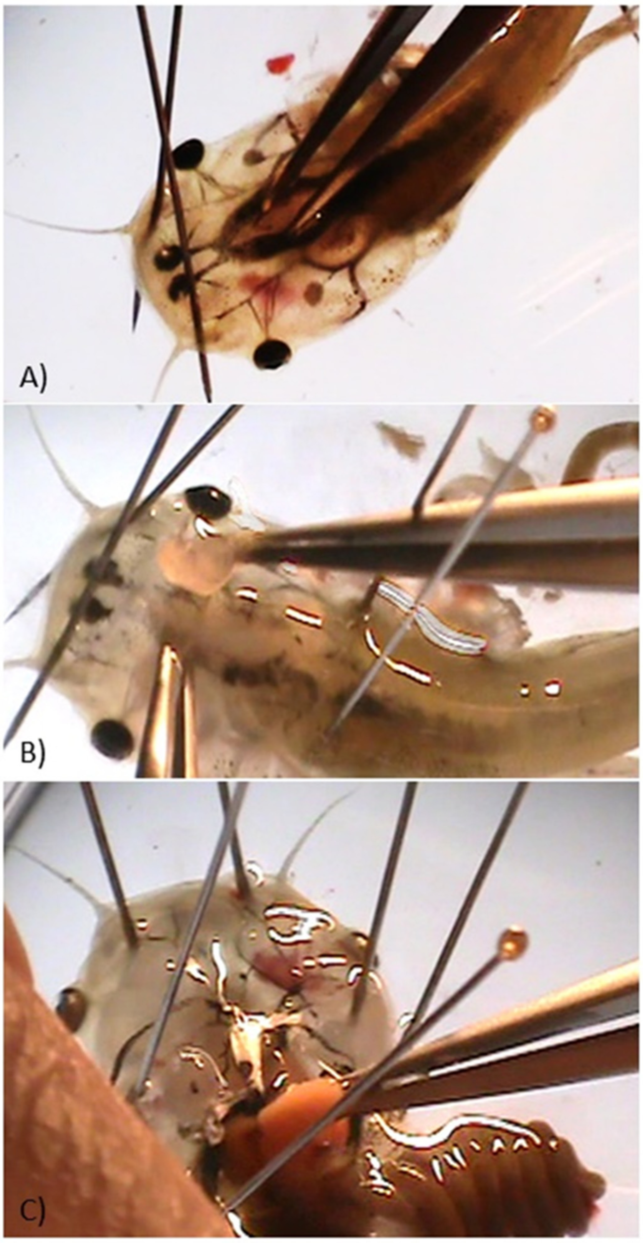

S. Figure S1. Dissecting sedated *Xenopus laevis* tadpoles at developmental stage NF 57. A) Location of incision point for brain removal is at forceps tips at the center of the head. B) Brain removed is a creamy white mass. C) Liver is removed ventrally, being yellowish brown and attached to the end of the gut.

On RNA extraction and rtPCR methods:

After preliminary investigations of handling and storing tissues using DNA/RNA Shield (Lot # ZRC182909; Cat # R1100-250, Zymo Research Corp, Irvine, CA) or 5% phenol/95% ethanol) (Phenol, Lot # 015842A, Cat # 0126-100G, Amresco LLC, Solon, OH; Ethanol is Aaper Alcohol and Chemical Co., Shelbyville, KY), livers and brains from adults were saved in DNA/RNA Shield for future gene expression work.

Supplementary Table S5

S Table S5. Statistical analyses[a] of incremental measures of total length of tadpoles (*Xenopus laevis*) exposed to neonicotinoids[b] compared to those in pesticide-free control media through 44 days post-hatch.

Treatment	Day 30 ANOVA	Day 44 ANOVA	MANOVA (Days 30 & 44)
THX 20 ppm	**<0.0001**	**0.0011**	**0.0003**
THX 100 ppm	*0.7297*	**0.0151**	**0.0265**
CLO 20 ppm	**0.0013**	*0.05*	**0.0093**
CLO 100 ppm	**0.0002**	**0.044**	**0.0008**

[a] ANOVA with post hoc Tukey's test for days 30 and 44. MANOVA longitudinal analysis for Days 30 &44.

[b] Thiamethoxam (THX) and clothianidin (CLO).

Supplementary Table S6

S Table S6. Results of Dunn's Test for pairwise comparisons of *Xenopus laevis* tadpole growth in the THX High (100 ppm) treatment group in examining whether the density of individuals contributed to any observed significant effects in length, weight, and developmental stage among the four replicates at the end of 44 days of exposure.

Comparison	Diff of Ranks	Q value	*P* value[a]	No. of Tadpoles showing difference
**Length**				
Hi3 vs Hi1	24.500	2.670	***0.046***	1
Hi3 vs Hi2	2.992	0.288	1.000	16
Hi3 vs Hi4	0.986	0.093	1.000	4
Hi4 vs Hi1	23.514	2.992	***0.017***	5
Hi4 vs Hi2	2.006	0.217	1.000	12
Hi2 vs Hi1	21.508	2.842	***0.027***	17
**Weight**				
Hi2 vs Hi1	21.469	2.837	***0.027***	17
Hi2 vs Hi4	2.944	0.319	1.000	12
Hi2 vs Hi3	1.583	0.152	1.000	16
Hi3 vs Hi1	19.885	2.167	0.181	1
Hi3 vs Hi4	1.361	0.128	1.000	4
Hi4 vs Hi1	18.524	2.357	0.110	5
**Stage**				
Hi2 vs Hi1	20.383	2.693	***0.042***	17
Hi2 vs Hi4	6.369	0.689	1.000	12
Hi2 vs Hi3	4.258	0.410	1.000	16
Hi3 vs Hi1	16.125	1.757	0.473	1
Hi3 vs Hi4	2.111	0.199	1.000	4
Hi4 vs Hi1	14.014	1.783	0.447	5

[a]Bold, italicized probability values indicate significance at α=0.05.

Supplementary Figure S2

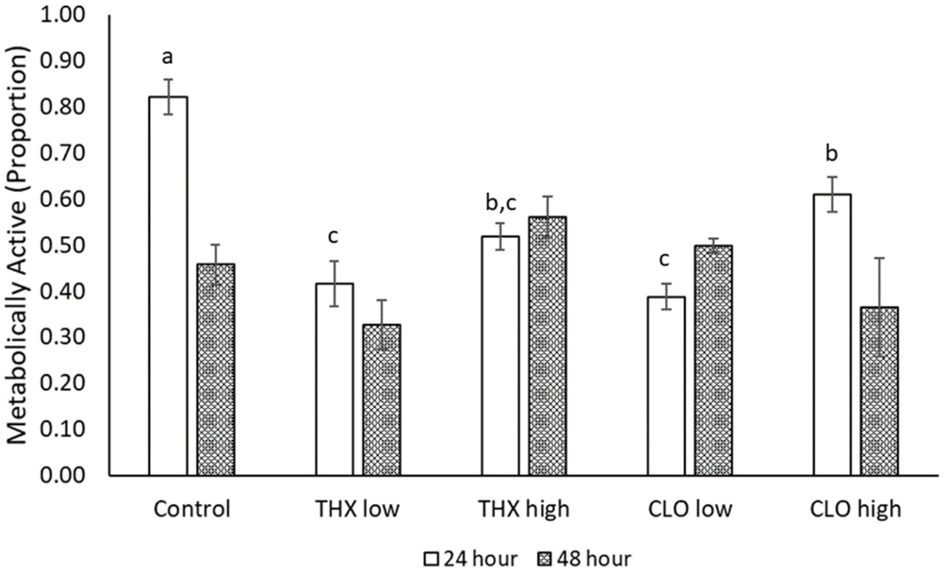

S. Figure S2. Flow cytometric data on the overall proportion of reductase activity from livers of *Xenopus laevis* having been exposed for 44 days in neonicotinoid-free control media, and thiamethoxam (THX) and clothianidin (CLO) treatment groups at 20 and 100 ppm, or low and high, respectively. Data were collected at 24 hours ($P < 0.0001$) and 48 hours ($P = 0.0717$) after tadpoles were removed from the treatment and placed in media. Data at 24 hours were averaged for analyses performed on four separate dates, with from 3 to 7 analyses per treatment group (n = 24 total analyses) by using 5 livers pooled per treatment. Data analysis at 48 hours were performed on two dates, with 2 analyses per treatment group (n = 10 total analyses).

Supplementary Figure S3

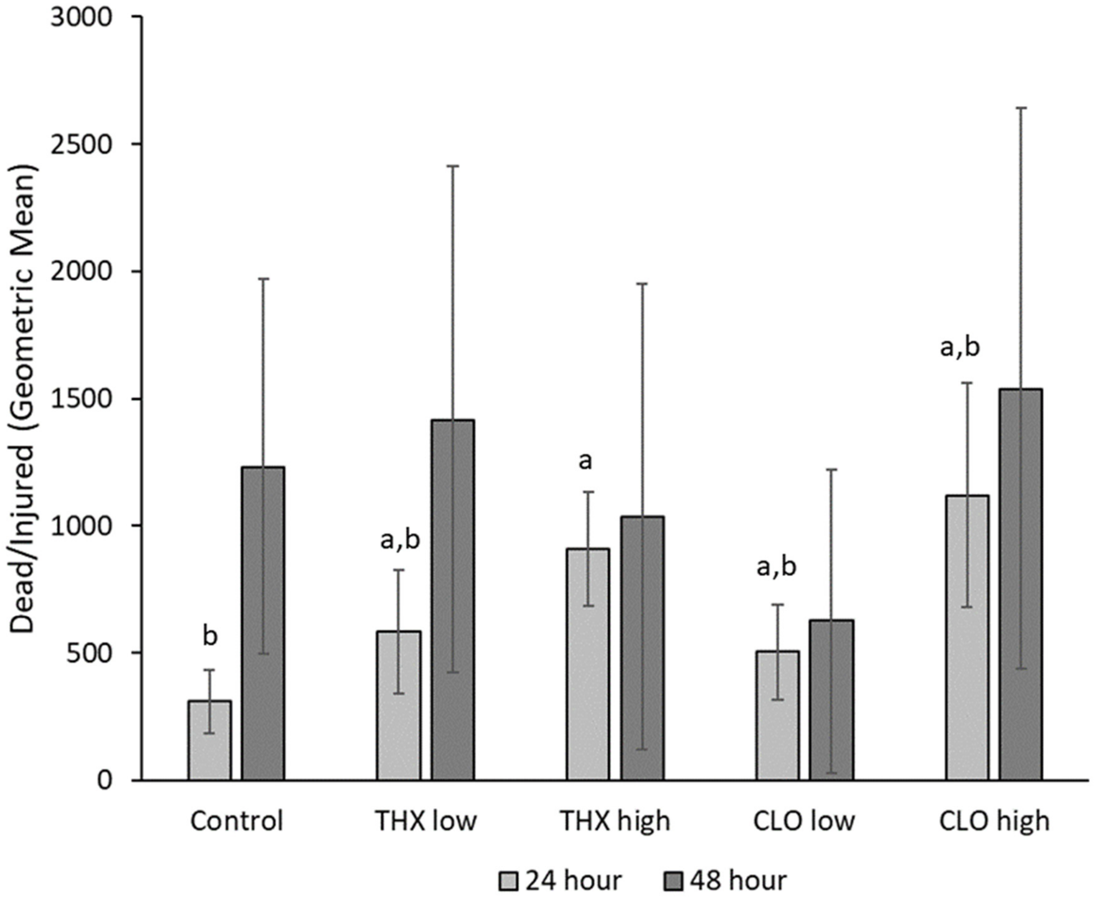

S Figure S3. Flow cytometric data on the cytotoxicity of the dead and injured cell populations livers of *Xenopus laevis* having been exposed for 44 days in neonicotinoid-free control media, and thiamethoxam (THX) and clothianidin (CLO) treatment groups at 20 and 100 ppm, or low and high, respectively. Geometric means of the flow cytometrically gated subpopulation was collected at 24 hours ($P = 0.0129$) and 48 hours ($P = 0.4212$) after tadpoles were removed from the treatments and placed in media. Data at 24 hours were averaged for analyses performed on four separate dates, with from 3 to 7 analyses per treatment group (n = 24 total analyses) by using 5 livers pooled per treatment. Data analysis at 48 hours were performed on two dates, with 2 analyses per treatment group (n = 10 total analyses).

Supplementary Figure S4

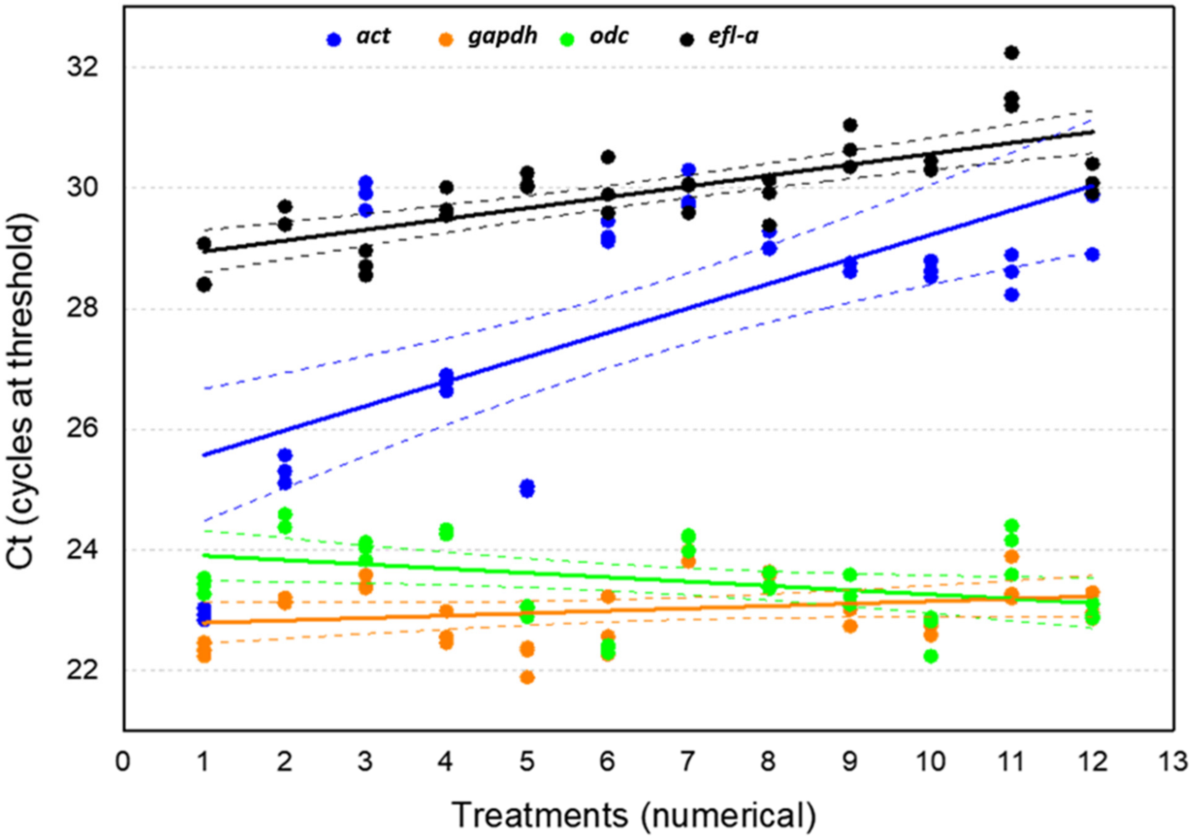

S Figure S4. The evaluation of potential reference genes by comparing thermal cycle threshold values by rtPCR. Fluorescent signals varied the least for ornithine decarboxylase (*odc*) and glyceraldehyde-3-phosphate dehydrogenase (*gapdh*), thus being selected as reference genes. Signals for actin (*act*) and elongation factor 1-α (*efl-a*) varied the most. Trend lines are plotted and confidence intervals are shown as dashed lines.

Supplementary Table S7

S Table S7. Multiple comparisons test results of *Xenopus laevis* growth parameters over 44 days of
exposure to thiamethoxam (THX) or clothianidin (CLO).

Treatment	Length (*P* value)	Weight (*P* value)	Developmental Stage (*P* value)
Control	*0.163*	*0.105*	*0.934*
THX 20 ppm	*0.227*	*0.832*	*0.889*
THX 100 ppm	**0.001**	**0.007**	**0.017**
CLO 20 ppm	*0.276*	*0.501*	*0.447*
CLO 100 ppm	*0.318*	*0.423*	*0.128*

[a]Testing was Holms-Bonferroni using Sigma Plot.

Any use of trade, firm, or product names is for descriptive purposes only and does not imply endorsement by the U.S. Government.
